# Multiparametric Renal Magnetic Resonance Imaging: Validation, Interventions, and Alterations in Chronic Kidney Disease

**DOI:** 10.3389/fphys.2017.00696

**Published:** 2017-09-14

**Authors:** Eleanor F. Cox, Charlotte E. Buchanan, Christopher R. Bradley, Benjamin Prestwich, Huda Mahmoud, Maarten Taal, Nicholas M. Selby, Susan T. Francis

**Affiliations:** ^1^Sir Peter Mansfield Imaging Centre, University of Nottingham Nottingham, United Kingdom; ^2^Centre for Kidney Research and Innovation, Royal Derby Hospital, University of Nottingham Derby, United Kingdom

**Keywords:** magnetic resonance imaging, hemodynamics, oxygenation, renal function, arterial spin labeling

## Abstract

**Background:** This paper outlines a multiparametric renal MRI acquisition and analysis protocol to allow non-invasive assessment of hemodynamics (renal artery blood flow and perfusion), oxygenation (BOLD T_2_^*^), and microstructure (diffusion, T_1_ mapping).

**Methods:** We use our multiparametric renal MRI protocol to provide (1) a comprehensive set of MRI parameters [renal artery and vein blood flow, perfusion, T_1_, T_2_^*^, diffusion (ADC, D, D^*^, f_p_), and total kidney volume] in a large cohort of healthy participants (127 participants with mean age of 41 ± 19 years) and show the MR field strength (1.5 T vs. 3 T) dependence of T_1_ and T_2_^*^ relaxation times; (2) the repeatability of multiparametric MRI measures in 11 healthy participants; (3) changes in MRI measures in response to hypercapnic and hyperoxic modulations in six healthy participants; and (4) pilot data showing the application of the multiparametric protocol in 11 patients with Chronic Kidney Disease (CKD).

**Results:** Baseline measures were in-line with literature values, and as expected, T_1_-values were longer at 3 T compared with 1.5 T, with increased T_1_ corticomedullary differentiation at 3 T. Conversely, T_2_^*^ was longer at 1.5 T. Inter-scan coefficients of variation (CoVs) of T_1_ mapping and ADC were very good at <2.9%. Intra class correlations (ICCs) were high for cortex perfusion (0.801), cortex and medulla T_1_ (0.848 and 0.997 using SE-EPI), and renal artery flow (0.844). In response to hypercapnia, a decrease in cortex T_2_^*^ was observed, whilst no significant effect of hyperoxia on T_2_^*^ was found. In CKD patients, renal artery and vein blood flow, and renal perfusion was lower than for healthy participants. Renal cortex and medulla T_1_ was significantly higher in CKD patients compared to healthy participants, with corticomedullary T_1_ differentiation reduced in CKD patients compared to healthy participants. No significant difference was found in renal T_2_^*^.

**Conclusions:** Multiparametric MRI is a powerful technique for the assessment of changes in structure, hemodynamics, and oxygenation in a single scan session. This protocol provides the potential to assess the pathophysiological mechanisms in various etiologies of renal disease, and to assess the efficacy of drug treatments.

## Introduction

Magnetic Resonance Imaging (MRI) offers the possibility to non-invasively assess the structure of the kidney as well as renal function in a single scan session. This article outlines the development of a quantitative functional multiparametric renal MRI protocol to probe hemodynamics (total and regional blood flow, perfusion), oxygenation [Blood Oxygen Level Dependent (BOLD) T_2_^*^ imaging], and microstructure (diffusion weighted imaging, longitudinal relaxation time T_1_ mapping) and describes associated analysis methods. This multiparametric MRI protocol is applied in healthy participants, to assess both reproducibility and the field strength dependence of MRI parameters between 1.5 and 3 Tesla (T). In addition, studies are performed in healthy participants to evaluate the possibility of using hypercapnia and hyperoxia to monitor changes in renal BOLD and T_1_ reactivity. Finally, pilot data demonstrating the feasibility of this multiparametric protocol in Chronic Kidney Disease (CKD) is shown.

The kidney is an intricate organ which regulates electrolytes, acid-base balance, and blood pressure and filters blood to remove water soluble waste products (Skorecki et al., 2016). Regulation of renal tissue oxygenation is complex, because renal blood flow is not only needed to prevent hypoxic injury but is inextricably linked to the requirement for glomerular filtration. The kidney's response to hypoxia cannot simply be an increase in renal blood flow, as this would also increase oxygen demand; a number of hemodynamic mechanisms are required to regulate the fine balance between oxygen delivery and consumption, and these can be measured using multiparametric MRI.

Oxygen delivery is determined by arterial blood flow, which is regulated by arterial blood pressure and intrarenal vascular resistance, local tissue perfusion and blood oxygen content (Evans et al., 2008). Arterial blood is supplied to the kidney via the renal artery, and blood flow can be estimated from phase contrast MR. The renal artery sequentially divides into segmental, interlobar, arcuate, and interlobular arteries before finally reaching the afferent arterioles that supply the glomeruli. The renal microcirculation varies depending on the location of each nephron within the cortex. In the outer cortex, glomerular efferent arterioles give rise to a capillary network that surrounds the tubules, important for reabsorption of water and electrolytes. In contrast, the efferent arterioles supply the medulla and give rise to the vasa recta, the long unbranched capillary loops that run into the inner medulla associated with the loop of Henle, as well as capillaries in the outer medulla. This facilitates concentration of urine in the medulla, but also has consequences for oxygenation.

The majority of arterial blood delivered to the kidney is directed toward the renal cortex, which primarily is responsible for filtration and tubular reabsorption; 5–15% is delivered to the medulla, whose purpose is concentration of the urine by maintaining a hypertonic environment. Despite the much lower proportional blood flow, the absolute blood flow to the medulla is still significant, reflecting large total renal blood flow (~20% of cardiac output), and a number of physiological and pathological conditions can produce significant redistribution of renal blood flow. However, a significant cortico-medullary oxygen gradient exists, with the inner medulla having a tissue oxygenation (pO_2_) as low as 10 mmHg, compared to 50 mmHg in the cortex. MR potentially provides a non-invasive method to assess this change in tissue oxygenation.

Tubular epithelial transport allows the kidney to regulate volume and composition of urine, but has significant energy and oxygen requirements. Reabsorption of sodium is the main determinant of oxygen consumption (Blantz et al., 2007; Thomson and Blantz, 2008), so that oxygen consumption is related to renal function, with reductions in glomerular filtration rate (GFR) resulting in a lower filtered load and lower requirement for sodium reabsorption. Renal perfusion is driven primarily by the need to maintain glomerular filtration rather than oxygenation, and therefore arteriovenous shunting of oxygen occurs to prevent hyperoxic tissue injury (via peri-glomerular shunts and between arterial and venous limbs of the vasa recta).

Although the kidney can reduce oxygen consumption in response to hypoxia, the lower pO_2_ in the medulla increases its propensity to ischemic damage, which is considered a key pathogenic event in acute kidney injury (AKI) and CKD (Venkatachalam et al., 2010). Since multiple interacting mechanisms operate in concert to provide tight regulation of intrarenal oxygenation, dysfunction of these mechanisms may contribute to the pathogenesis of kidney disease. For example, vascular morphologic changes may occur such as, capillary rarefaction as well as factors that affect regional blood flow and oxygen diffusion e.g., upregulation of the renin-angiotensin or sympathetic nervous systems (Adler et al., 2004). Alternatively, other changes may impact regional oxygen utilization such as alterations in global and single nephron GFR, drugs interfering with glomerular hemodynamics or tubular transport and hydration status. In addition to the complexity of kidney function, renal diseases such as CKD are diverse in terms of pathophysiological processes, etiology and outcomes, highlighting the need for multiparametric MRI measures. Renal perfusion and tissue oxygenation appear central integrating factors in kidney disease, highlighting the need to perform a combined assessment of these parameters, and regardless of the nature of initial insult, fibrosis is the final common pathway.

The potential use of complementary MRI techniques to non-invasively assess multiple parameters to provide a wealth of information on renal blood flow and regional perfusion, tissue oxygenation, and degree of fibrosis, as well as behavior in low or high oxygen or carbon dioxide, will undoubtedly aid the understanding of kidney disease. Prior to the use of MRI in kidney disease, the reproducibility of MRI measures and their dependence on different factors must be understood in normal tissue.

Here, we assess the inter-subject variability, repeatability, and field strength dependence of multiparametric MRI measures in healthy participants. Physiological modulations such as, hyperoxia and hypercapnia are performed, and pilot data are shown to illustrate the feasibility of detecting changes in MR measures in CKD.

## Materials and methods

### Study design

Studies were carried out according to the principles of the Declaration of Helsinki. Healthy participant studies were approved by the Local Ethics Committee and patient studies were approved by the East Midlands Research Ethics Committee. Written informed consent was obtained from all participants.

Imaging was performed on either a 1.5 or 3 T Philips whole body MR scanner. Data is presented from studies that use the multiparametric renal MRI protocol, comprising quantification of renal blood flow and perfusion, renal oxygenation, and markers of renal microstructural change due to fibrosis/inflammation. All data was collected with subjects fasted for at least 2 h prior to their scan.

#### Variability, repeatability, and field strength dependence in healthy participants

Here, we evaluate the variation in MRI measures within normal tissue of a healthy participant cohort, specifically we assess renal artery and renal vein blood flow [as measured with phase contrast (PC)-MRI], kidney perfusion [as measured with arterial spin labeling (ASL)], T_1_ measures [and a comparison of readout schemes: spin echo–echo planar imaging (SE-EPI) and balanced fast field echo (bFFE)], tissue oxygenation (from BOLD T_2_^*^), diffusion weighted imaging (DWI), and total kidney volume. Data collated across a number of studies are first shown, giving a cohort of 127 participants (88 male) with mean age of 41 ± 19 years. This data is then divided into two groups comprising healthy participants <40 years and >40 years [see Section Application in Chronic Kidney Disease (CKD)]. In addition the field strength dependence of MR relaxation times is assessed. Since clinical MR scanners at both 1.5 and 3 T are now widely available, the field dependence of MR relaxation time measures of T_1_ (using both SE-EPI and bFFE) and T_2_^*^ for renal cortex and renal medulla was assessed.

A subset of 11 participants (age 20–28 years, body mass index 20–26 kg/m^2^) had two/three repeat 3 T scans at the same time of day and after an overnight fast to limit diurnal and dietary variability. To determine the between session repeatability of MRI measures, the intra-subject Coefficient of Variation (CoV; defined as the standard deviation/mean) and intra class correlation (ICC, average measures, two-way random, absolute agreement) were assessed.

#### Physiological modulation in healthy participants

Physiological modulations, such as gas enrichment by hypercapnia, hyperoxia, or carbogen (hypercapnic-hyperoxia; Milman et al., 2013) may provide a more sensitive marker to assess changes in renal oxygenation and microcirculation reactivity and functionality, and changes in these parameters associated with pathology. Here, we assess the change in MRI parameters in healthy participants in response to hypercapnia and hyperoxia. We induced hypercapnia and hyperoxia using a sequential gas delivery breathing circuit and a prospective, feed-forward gas delivery system (Respiract™, Thornhill Research Inc., Toronto, Canada) to control and monitor end-tidal oxygen (P_ET_O_2_) and carbon dioxide (P_ET_CO_2_) partial pressures. Hypercapnia was targeted at P_ET_CO_2_ ~6 mmHg above the subjects' baseline value whilst keeping P_ET_O_2_ constant at the subjects' resting value, the paradigm comprised 5 min normoxia and 5 min of hypercapnia. Hyperoxia was targeted at P_ET_O_2_ ~500 mmHg with P_ET_CO_2_ targeted to remain constant at the subjects' resting value. The paradigm comprised 5 min normoxia and 5 min of hyperoxia, P_ET_O_2_ was increased/decreased over a 1 min transition period.

BOLD T_2_^*^ was measured at 3 T using a multi-gradient echo Fast Field Echo (mFFE) sequence in six healthy participants (3 male, mean age 25 years, range 22–28 years) during the hyperoxia and hypercapnia challenge. T_1_ was measured during a hyperoxic challenge in five healthy participants (3 male, mean age 26 years, range 22–31 years) using an inversion recovery sequence with modified respiratory triggering and a bFFE readout at 3 T.

#### Application in chronic kidney disease (CKD)

To demonstrate the feasibility of use of the multiparametric MR protocol in patients, 11 patients with CKD Stage 3 or 4 were scanned (inclusion criteria: estimated GFR 15–66 ml/min/1.73 m^2^, age 18–85 years). Baseline blood pressure and estimated GFR of the patients was recorded. The complete multiparametric protocol was performed comprising of localizer scans, PC-MRI, ASL, T_1_, T_2_^*^, and DWI data as described below. All scans were acquired in approximately 45 min.

### The multiparametric MRI protocol

**Figure 1:**
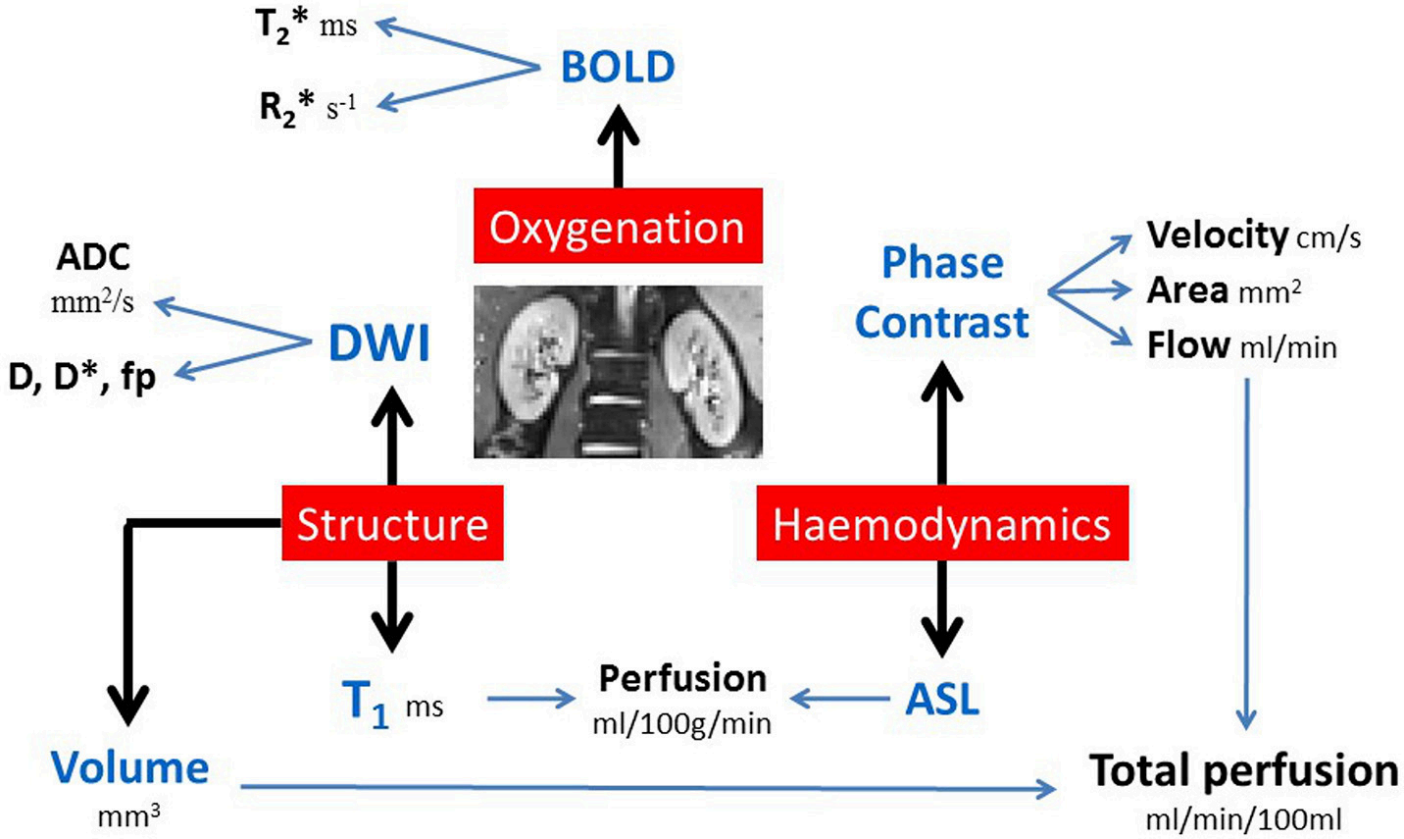
Multiparametric non-invasive renal MRI protocol.

Figure 1 outlines the key MRI parameters within the multiparametric protocol, these measures can all be performed within a 45 min scan session. All mapping data are collected with matched geometry with slices in a coronal-oblique plane through the long axis of the kidneys, allowing automated interrogation of the resulting multiparametric maps. All data is acquired using respiratory triggering or an end-expiration breath hold to ensure data is acquired at the same point in the respiratory cycle. Each of the parameters within this protocol are outlined below.

#### Localizers and kidney volume assessment

Balanced turbo field echo (bTFE) scans are acquired in three orthogonal planes (30 slices of 1.75 × 1.75 × 7 mm^3^ resolution, data collected in single breath hold per orientation). These scans provide a localizer to allow accurate planning of subsequent images, and segmentation of these images yields total kidney volume.

#### Phase contrast (PC)-MRI to assess renal artery and vein blood flow

Prior to the PC-MRI acquisition, an angiogram is acquired to plan the placement of the PC-MRI renal artery slice to ensure that it is positioned prior to any bifurcations of the artery.

PC-MRI is then used for the measurement of blood flow in the renal arteries and veins (Debatin et al., 1994; Schoenberg et al., 1997; Bax et al., 2005; Park et al., 2005; Dambreville et al., 2010). PC-MRI is performed using a single slice TFE image placed perpendicular to the vessel of interest. Multiple phases are collected across the cardiac cycle when imaging the renal artery (20 phases) and renal vein (15 phases). Imaging parameters use a flip angle of 25°, reconstructed resolution 1.2 × 1.2 × 6 mm^3^, and velocity encoding of 100/50 cm/s for renal artery and vein, respectively. Each measurement is acquired during a single 15–20 s breath hold, dependent on the subjects' heart rate.

#### Arterial spin labeling (ASL) to assess renal cortex perfusion

ASL uses magnetically labeled water protons in blood that act as a diffusible tracer, providing an internal endogenous contrast. By subtracting labeled images (radiofrequency magnetic labeling) from control images (no labeling applied), perfusion maps can be quantified using a kinetic model (Buxton et al., 1998). Renal tissue perfusion assessed by ASL has been implemented in healthy (Karger et al., 2000; Martirosian et al., 2004; Boss et al., 2005; Kiefer et al., 2009; Gardener and Francis, 2010; Cutajar et al., 2012, 2014; Park et al., 2013; Gillis et al., 2014; Tan et al., 2014; Hammon et al., 2016), transplanted (Artz et al., 2011a,b; Niles et al., 2016) and diseased (Michaely et al., 2004; Boss et al., 2005; Fenchel et al., 2006; Ritt et al., 2010; Rossi et al., 2012; Dong et al., 2013; Heusch et al., 2013; Tan et al., 2014) kidneys.

To measure renal cortex perfusion, we have implemented a respiratory-triggered FAIR (Flow-sensitive Alternating Inversion Recovery) ASL scheme. For imaging, we use either a SE-EPI or bFFE readout. Typical imaging parameters at 3 T are a post label delay (PLD) time of 1,800 ms (depending on choice of readout scheme and field strength Buchanan et al., 2015), 40 label/control pairs, 288 × 288 mm field of view, 3 × 3 × 5 mm^3^ voxel resolution. A SE-EPI readout provides good spatial coverage, allowing multiple slices to be acquired in a short acquisition time over the ASL signal curve (five slices in ~ 300 ms at 3 T). A bFFE readout provides the benefit of high spatial resolution, typically 1.5 mm in-plane spatial resolution and 5 mm slice thickness, however this can limit slice coverage due to the increased acquisition time per slice (~ 250 ms slice spacing at 3 T). Since ASL is a subtraction technique, we use respiratory triggering to minimize the effects of respiratory motion leading to misalignment or blurring. It is important to take into account the arrival time of the blood to the tissue when quantifying perfusion, particularly in disease where the arrival time can be increased, resulting in an apparent reduction in perfusion. A separate scan to assess the arrival time of the blood to the tissue is acquired, by collecting ~4 label/control pairs at shorter PLD times (500, 700, 900, 1,100 ms). A base equilibrium M_0_ scan and T_1_ map are also required for accurate perfusion quantification. Depending on respiratory rate, scan time for 40 label/control pairs of ASL data is approximately 6 min, with a further 2 min for assessment of arrival time and collection of a base M_0_ scan.

#### Longitudinal relaxation time T_1_ mapping

The assessment of the longitudinal relaxation time T_1_ of tissue is essential for the quantification of ASL perfusion. Recently, T_1_ mapping alone has been shown to provide an important parameter by which to evaluate fibrosis (due to the association of collagen with supersaturated hydrogel) or inflammation (interstitial edema, cellular swelling). T_1_ has been shown to correlate well with fibrosis and edema in the myocardium (Iles et al., 2008; Jellis and Kwon, 2014), liver (Hoad et al., 2015; Tunnicliffe et al., 2017), and more recently in the kidney (Friedli et al., 2016).

**Figure 2:**
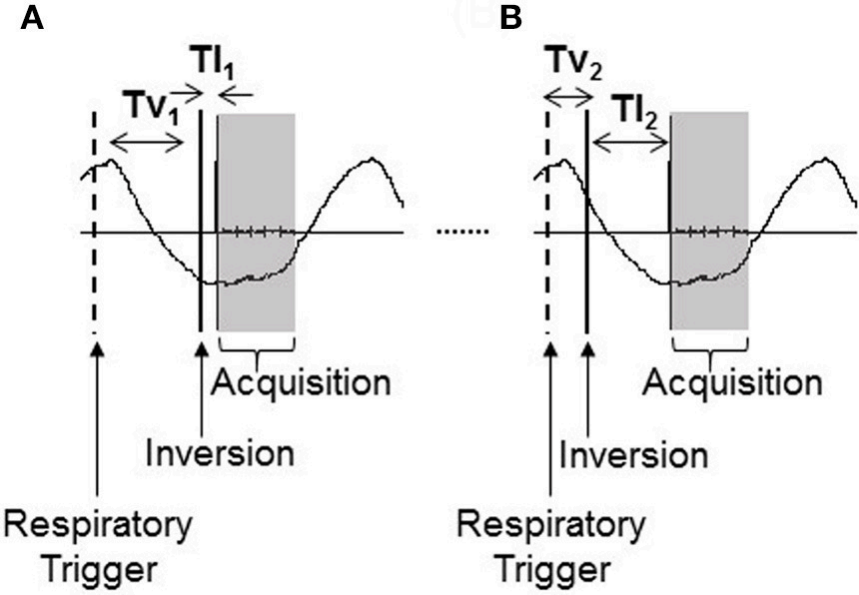
Modified respiratory triggered inversion recovery sequence shown for **(A)** short (TI_1_) and **(B)** long (TI_2_) inversion time. By altering the variable delay, Tv, each image acquisition is collected at a constant time (Tv + TI) following the respiratory trigger. First arrow indicates the respiratory trigger (“Respiratory Trigger”), second the inversion pulse (“Inversion”), and the shaded block indicates the image acquisition readout (“Acquisition”).

Here, an inversion recovery sequence with a modified respiratory triggering scheme (Figure 2) has been developed to minimize respiratory-induced abdominal motion between images of differing contrast collected across the range of inversion times required to compute a T_1_ map. The respiratory trigger is applied at the peak of inspiration in the respiratory cycle and the image is then acquired at a constant time following this trigger, during the flat end-expiration period of the respiratory cycle. A variable delay, Tv, is introduced between the respiratory trigger and the inversion pulse which is followed by the inversion time, TI, between the inversion pulse and image acquisition. By holding the total time period Tv + TI constant, this results in all image readouts for all inversion times being collected at a constant time of (Tv + TI) following the respiratory trigger and as such all images are aligned across the inversion times. The time (Tv + TI) is chosen to be at the end-expiration period of the respiratory cycle to minimize any potential motion artifacts.

Here, we use either a SE-EPI or bFFE readout scheme for T_1_ mapping. In general, the same readout scheme as is used for the ASL acquisition is chosen. Importantly, the chosen image readout scheme has an impact on the measured T_1_ value. A SE-EPI readout scheme provides a “true” T_1_ value, whereas a bFFE readout scheme results in an “apparent” T_1_, shorter than the “true” T_1_ due to the influence of transverse relaxation rates (T_2_/T_2_^*^; Schmitt et al., 2004). At 3 T, we typically collect 13 inversion times of 200, 300, 400, 500, 600, 700, 800, 900, 1,000, 1,100, 1,200, 1,300, and 1,500 ms in a total scan time of <3 min. For a multi-slice bFFE readout, the temporal slice spacing is longer (~250 ms at 3 T) than for a SE-EPI (~60 ms at 3 T) readout, and therefore the dynamic range of TIs can be increased by acquiring the scans ascend, descend and interleaved slice order.

An alternative scheme for T_1_ mapping is to use a modified look-locker inversion recovery (MOLLI) sequence originally developed for cardiac T_1_ mapping. This typically involves the acquisition of a cardiac-gated single-shot MOLLI sequence using a bFFE readout [23] with a 3(3)3(3)5 sampling pattern collected in a breath hold. However, this acquisition scheme is not best suited to the kidney, since it is cardiac triggered, requires a number of breath holds for complete coverage of the kidneys, and does not match the ASL acquisition readout scheme, and so it is not implemented in our multiparametric protocol.

#### Diffusion weighted imaging (DWI)

DWI assesses the thermally induced Brownian motion of water within tissues, which can be quantified from the Apparent Diffusion Coefficient (ADC). ADC may also be affected by factors such as tubular flow and capillary perfusion, which can be better distinguished using the IntraVoxel Incoherent Motion (IVIM) model to quantify pure diffusion (D; Le Bihan et al., 1988). In DWI, at least two single-shot echo-planar images are acquired without and with diffusion weighting gradients (b-values) from which molecular diffusion can be quantified and spatially mapped. It is important to note that the quantification of the ADC is affected by the b-values acquired. In this multiparametric protocol, DWI data is acquired with a SE-EPI readout at multiple b-values (for example, 11 b-values of 0, 5, 10, 20, 30, 50, 100, 200, 300, 400, 500 s/mm^2^). The highest b-value is chosen such that the echo time (TE) does not become so long as to limit the signal-to-noise ratio (SNR) of the image. Typically a 288 × 288 mm field of view is used with 3 × 3 × 5 mm^3^ voxel resolution which has a minimum TE of 56 ms. This sequence is acquired with respiratory triggering such that the image readouts are collected at the end-expiration period. For 11 b-values, the acquisition time is approximately 8 min.

#### Blood oxygenation level dependent (BOLD) imaging to assess tissue oxygenation

BOLD MRI exploits the paramagnetic properties of deoxygenated blood, which acts to shorten the transverse relaxation time constant (T_2_^*^)—alternatively expressed as the relaxation rate R_2_^*^ (1/T_2_^*^)—a measure which provides an indirect non-invasive assessment of oxygen content. Higher R_2_^*^ (or lower T_2_^*^) is an indicator of lower tissue pO_2_. BOLD MRI is more sensitive at detecting changes in medullary compared to cortical pO_2_ due to their relative positions on the oxygen dissociation curve—cortical pO_2_ lies near the plateau of the hemoglobin oxygenation curve and medullary pO_2_ lies on the linear part of the curve, thus a large change in local pO_2_ is needed to cause a similar change in R_2_^*^ for the cortex compared to the medulla. The use of BOLD MRI to measure renal oxygenation has been extensively studied. However, it should be highlighted that a number of other factors, such as, hydration status, dietary sodium intake, and susceptibility effects also alter BOLD R_2_^*^ (Pruijm et al., 2017), this can make it difficult to draw definite conclusions from its independent use. For a review of this technique, see Pruijm et al. (2017). In this multiparametric protocol, BOLD T_2_^*^ data is acquired using a mFFE sequence with multiple slices. Typical imaging parameters are 1.5 mm in-plane resolution, 5 mm slice thickness, initial TE 5 ms, TE spacing 3 ms, 12 echoes, flip angle 30°. Each measurement is acquired in a single ~17 s breath hold.

### Analysis of multiparametric MRI

#### Kidney volume assessment

Analyze9 software (AnalyzeDirect, Overland Park, KS) is used to define a region of interest around the kidneys on each bTFE localizer image slice. Total kidney volume can then be calculated by summing across all slices, typically the coronal slices are used for organ volume measures. Analysis time is approximately 10 min.

#### PC-MRI renal blood flow assessment

A region of interest is placed over the vessel using Q-flow software (Philips Medical Systems, Best, NL). Mean flow velocity (cm/s), mean cross-sectional area of the lumen (mm^2^), and hence mean bulk renal blood flow (ml/s) over the cardiac cycle, are calculated for each vessel. Total perfusion of each kidney can then be calculated by correcting the renal blood flow to kidney volume. Analysis time is approximately 2 min per vessel.

#### Multiparametric interpretation

Combining multiparametric MRI maps adds considerable insight into the underlying physiology. We have developed a multiparametric image analysis program (MATLAB, The Mathworks Inc., Natick, MA) that generates and combines the parametric ASL perfusion, T_1_, diffusion, and BOLD T_2_^*^ maps in the same data space. The multiparametric maps can then be used to perform multivariate analysis of structural and hemodynamic measures in automated regions of interest in the cortex and medulla.

##### Mapping perfusion from ASL data

Individual perfusion weighted difference images (control-label) are calculated, inspected for motion (exclude >1 voxel movement) or realigned, and averaged to create a single perfusion-weighted (ΔM) map. ΔM, T_1_ maps (see below), and M_0_ maps are then used in a kinetic model (Equation 1; Buxton et al., 1998) to calculate tissue perfusion (*f*) maps (in ml/100 g tissue/min). T_1,blood_ is assumed to be 1.55 s at 3 T and 1.36 s at 1.5 T (Dobre et al., 2007), whilst λ, the blood-tissue partition coefficient, is assumed to be 0.8 ml/g for kidney. Analysis time is approximately 10 min.

$$\begin{array}{l} \Delta M\left(PLD\right)=2M_{0}\frac{f}{\lambda }\frac{e^{PLD/T_{1,app-e}\;PLD/T_{1,blood}}}{1/T_{1,blood}-1/T_{1,app}}\text{ where} \end{array}$$

(1) $$\begin{array}{l} 1{/}_{T_{1,app}}=\;1{/}_{T_{1}}+f{/}_{\lambda } \end{array}$$

##### Longitudinal relaxation time (T_1_) mapping

Inversion recovery data is fit on a voxel-by-voxel basis to Equation (2) to generate a “true” T_1_ map for the SE-EPI readout, for the bFFE readout an “apparent” T_1_ map is obtained. Analysis time is approximately 3 min of user intervention, and up to 1 h processing time on a standard pc.

(2) $$\begin{array}{l} S\left(\text{TI}\right)=S_{0}\left(1-2e^{{-}^{\text{TI}}{/}_{T_{1}}}\right) \end{array}$$

##### Mapping ADC, D, D^*^, and f_*p*_ from DWI data

DWI data are fit to form ADC maps (in mm^2^/s) by taking the log of the exponential signal decay (Equation 3). In addition, since the DWI data is collected at a number of b-values, it is possible to model the bi-exponential IVIM model (Equation 4). In the IVIM model, D (in mm^2^/s) is the pure tissue molecular diffusion coefficient representing the diffusion coefficient of slow or non-perfusion-based molecular diffusion, D^*^ (in mm^2^/s) is the pseudodiffusion coefficient which is the fast or perfusion-based molecular diffusion representing intravoxel microcirculation or perfusion, and *f*_*p*_ is the perfusion fraction (%) of the voxel (Le Bihan et al., 1988; Koh et al., 2011). To fit data to the IVIM model, D was first fit to Equation (3) for b-values of >200 s/mm^2^, this assumes that the pseudodiffusion component D^*^ can be neglected above this value. Second, *f*_*p*_ was determined from the zero intercept of this fit. Finally, D^*^ was obtained from the monoexponential fit using the precalculated values of D and *f*_*p*_ (Suo et al., 2015). Analysis time is approximately 5 min of user intervention, and up to 5 min processing time on a standard pc.

(3) $$\begin{array}{l} S\left(b\right)=\;S_{0}e^{-b.ADC} \end{array}$$

(4) $$\begin{array}{l} S\left(b\right)=\;f_{p}S_{0}e^{-b.D^{\text{*}}}+\left(1-f_{p}\right)S_{0}e^{-b.D} \end{array}$$

##### BOLD MRI to map T_2_^*^/R_2_^*^

mFFE data are fit voxelwise using a weighted echo time (TE) fit to form T_2_^*^/R_2_^*^ maps from the log of the exponential signal decay (Equation 5). Analysis time is approximately 5 min.

(5) $$\begin{array}{l} S(TE)=\;S_{0}e^{{-}^{TE/{T_{2}}^{*}}} \end{array}$$

##### Interpretation of multiparametric maps

**Figure 3:**
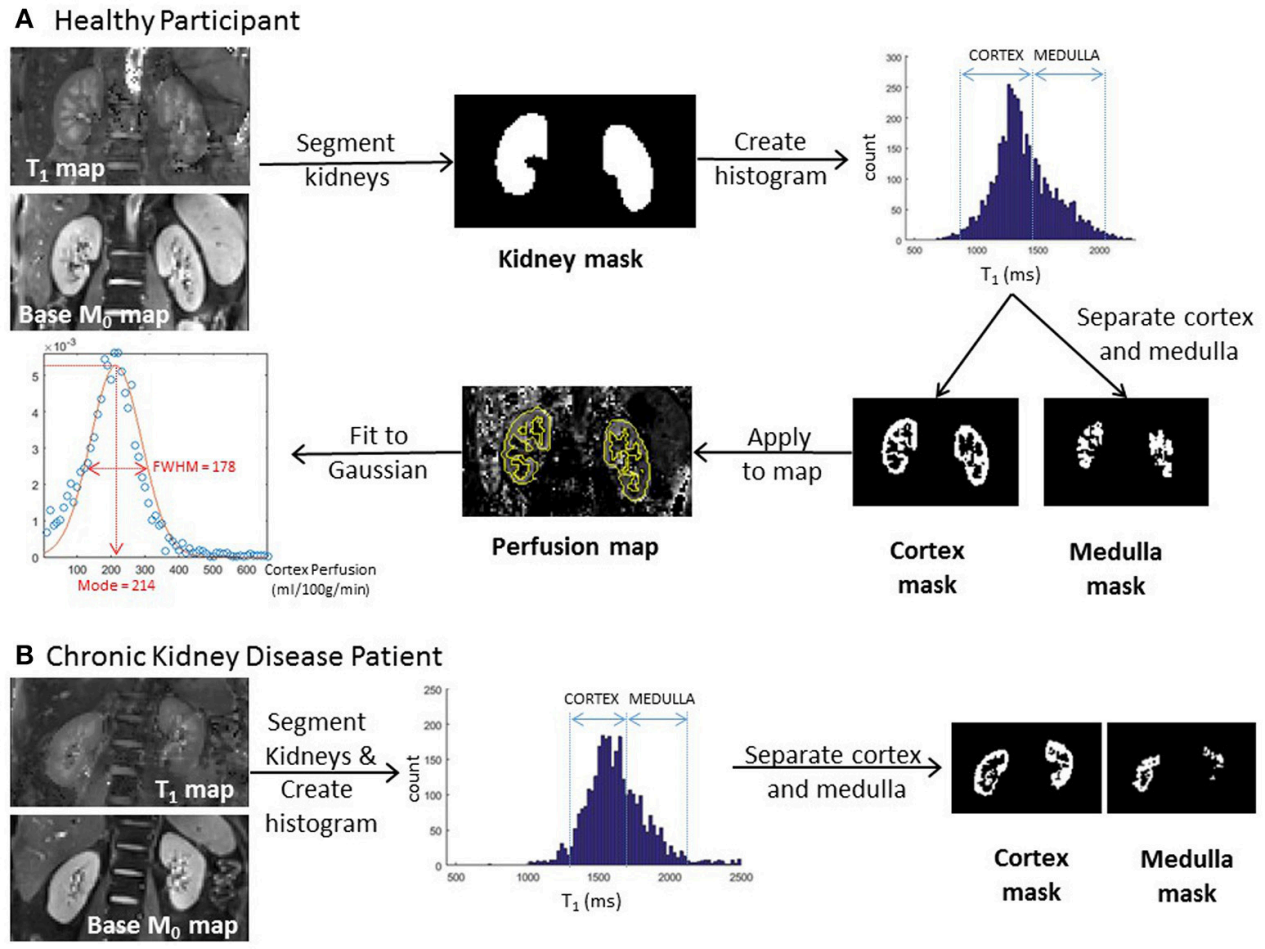
**(A)** Example image analysis for a healthy participant indicating segmentation of the kidneys from the T_1_ map, definition of cortex and medulla masks from the histogram, and the application of the renal cortex mask to an arterial spin labeling perfusion map allowing the interrogation of a histogram [for mode and full-width-at-half-maximum (FWHM)] of renal cortex perfusion values. **(B)** Example image analysis for a chronic kidney disease patient indicating definition of cortex and medulla masks from the T_1_ histogram of the kidneys.

Binary whole kidney masks are formed from the manual segmentation of the base equilibrium M_0_ scan or T_1_ map. To distinguish renal cortex and medulla, a histogram of T_1_ values across both kidneys is formed (with a bin size of 20 ms). Two peaks in the histogram, originating from the renal cortex and medulla, can be identified from which to form separate renal cortex and renal medulla masks. This segmentation procedure is illustrated for both a healthy participant and CKD patient in Figure 3. It should be noted that T_1_ values are elevated in CKD [see Section Application in Chronic Kidney Disease (CKD)], but sufficient corticomedullary differentiation remains to segment the cortex from medulla. These binary cortex and medulla masks can then be applied to each parametric map (perfusion, T_1_, ADC, D, D^*^, and *f*_*p*_) to interrogate identical regions of interest in which to assess mean values of each parameter. Importantly, to assess heterogeneity of measures and remove bias, a Gaussian curve fit can be applied to the histogram to determine both the mode and full-width-at-half-maximum (FWHM) of renal cortex and medulla parameter values across one or both kidneys (Rossi et al., 2012). The assessment of corticomedullary differentiation (medulla-cortex) in MRI parameters also provides important information. Analysis time is approximately 10 min.

## Results

All results given are the mean and standard deviation across participants.

### Variability, repeatability, and field strength dependence in healthy participants

**Figure 4:**
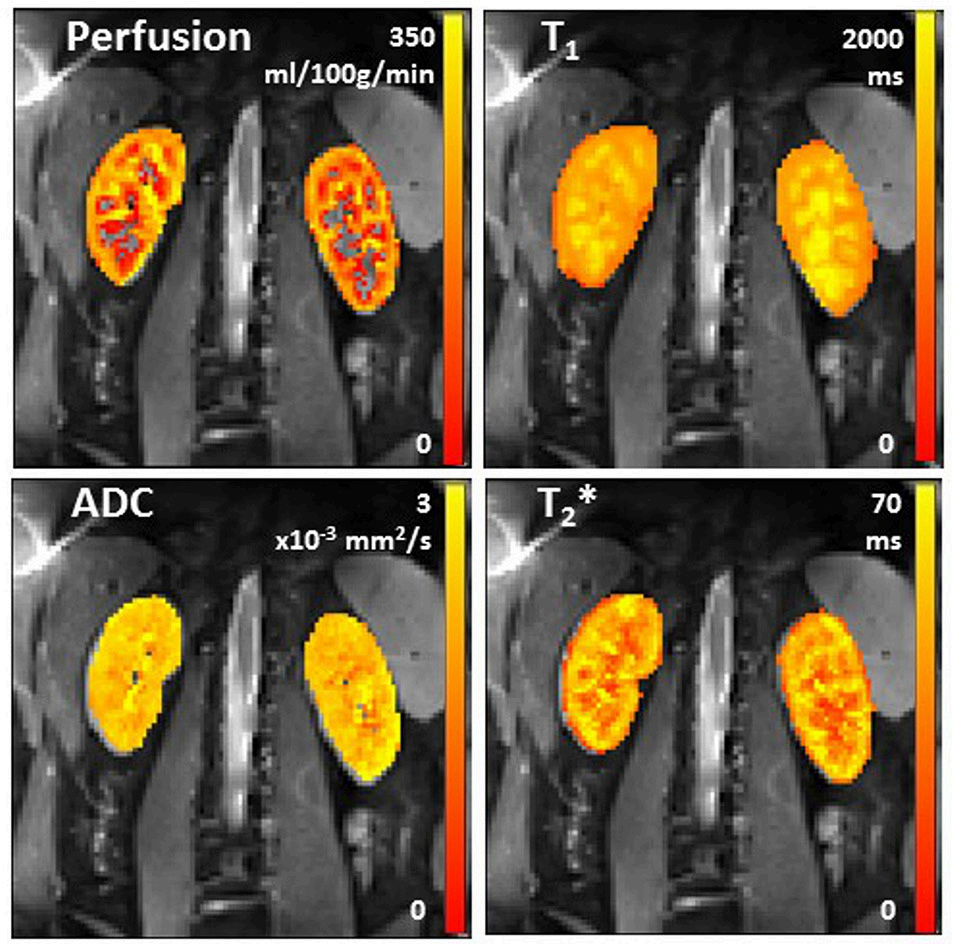
Example arterial spin labeling perfusion, longitudinal relaxation time T_1_, ADC (apparent diffusion coefficient), and transverse relaxation time T_2_* maps in a healthy participant.

Figure 4 shows example multiparametric MRI maps for a single healthy participant collected at 3 T, illustrating that the maps can be combined in the same data space and allow assessment of heterogeneity across the kidney. Table 1 provides the mean and associated standard deviation for MRI parameters collected across the cohort of healthy participants, with the number of subjects included in each analysis provided, and a comparison to literature values.

**Table 1:** Between-subject variability for multiparametric MRI measures in healthy participants and associated literature values.

**Parameter**		**Between subject variability**		**Literature values**
		**Mean ± std. dev**.	**Number of subjects**	
Single renal artery flow		373 ± 105 ml/min	73	583 ± 164 ml/min (Bax et al., 2005)
				443 (404–481) ml/min (Khatir et al., 2015)
				365 ± 119 ml/min (Khatir et al., 2014)
				0.48 ± 0.13 L/min (Steeden and Muthurangu, 2015)
Single renal vein flow		410 ± 134 ml/min	28	
Total perfusion to single kidney		222 ± 60 ml/min/100 ml	11	3.6 (3.2–4.0) ml/min/cm^3^ (Khatir et al., 2015)
Cortex perfusion		255 ± 70 ml/100 g/min	85	204 ml/min/100 g (Cutajar et al., 2012)
				355 ± 71 ml/100 g/min (Gardener and Francis, 2010)
				321 ± 63 ml/min/100 g (Gillis et al., 2014)
				200–260 ml/100 g/min (Martirosian et al., 2004)
				367 ± 41 ml/100 g/min (Wang et al., 2012)
Cortex T_1_(at 3 T)	SE-EPI	1367 ± 79 ms	21	1,376 ± 104 ms (MOLLI) (Gillis et al., 2014)
	bFFE	1124 ± 114 ms	26	1,142 ± 154 ms (FSE) (de Bazelaire et al., 2004)
Medulla T_1_(at 3 T)	SE-EPI	1655 ± 76 ms	20	1,651 ± 86 ms (MOLLI) (Gillis et al., 2014)
	bFFE	1389 ± 126 ms	25	1,545 ± 142 ms (FSE) (de Bazelaire et al., 2004)
Cortex T_2_^*^ (at 3 T)		49.6 ± 6.6 ms (R_2_^*^ 20.6 ± 3.3 s^−1^)	18	51 ± 8 ms (Ding et al., 2013)
				21.8 ± 1.2 s^−1^ (Li et al., 2004b)
				11.1 ± 3.8 s^−1^ (Park et al., 2012)
				18.2 ± 1.7 s^−1^ (Piskunowicz et al., 2015)
				17.4 ± 1.1 s^−1^ (van der Bel et al., 2016)
Medulla T_2_^*^ (at 3T)		29.7 ± 5.4 ms (R_2_^*^ 34.9 ± 6.9 s^−1^)	18	37.4 ± 1.2 s^−1^ (Li et al., 2004b)
				36 ± 7 ms (Ding et al., 2013)
Cortex ADC		2.3 ± 0.3 × 10^−3^ mm^2^/s	39	2.4 ± 0.1 × 10^−3^ mm^2^/s (Zhang et al., 2010)
				2.63 ± 0.08 × 10^−3^ mm^2^/s (Cutajar et al., 2011)
				2.4 ± 0.2 × 10^−3^ mm^2^/s (Sigmund et al., 2012)
				2.00 ± 0.07 × 10^−3^ mm^2^/s (Thoeny et al., 2005)
				2.4 ± 0.1 × 10^−3^ mm^2^/s (Wittsack et al., 2010)
Cortex D		1.7 ± 0.3 × 10^−3^ mm^2^/s	38	1.8 ± 0.1 × 10^−3^ mm^2^/s (Zhang et al., 2010)
				1.96 ± 0.09 × 10^−3^ mm^2^/s (Sigmund et al., 2012)
				1.5 ± 0.1 × 10^−3^ mm^2^/s (Wittsack et al., 2010)
				2.44 ± 0.12 × 10^−3^ mm^2^/s (Notohamiprodjo et al., 2015)
Cortex D^*^		10.7 ± 4.5 × 10^−3^ mm^2^/s	29	14.2 ± 0.8 × 10^−3^ mm^2^/s (Zhang et al., 2010)
				24.56 ± 6.10 × 10^−3^ mm^2^/s (Sigmund et al., 2012)
				13.1 ± 2.2 × 10^−3^ mm^2^/s (Wittsack et al., 2010)
				22.7 ± 10.6 × 10^−3^ mm^2^/s (Notohamiprodjo et al., 2015)
Cortex *f_*p*_*		28 ± 10%	29	31 ± 2% (Zhang et al., 2010)
				18.7 ± 3.5% (Sigmund et al., 2012)
				52 ± 10% (Wittsack et al., 2010)
				26.6 ± 6.1% (Notohamiprodjo et al., 2015)
Total kidney volume		367 ± 58 ml (Mean 184 ± 29 ml)	22	Mean across kidneys:
				141.6 ± 28.5 ml (Seuss et al., 2017)
				167 (97–307) ml (Cohen et al., 2009)
				196 (136–295) ml (van den Dool et al., 2005)

T_1_, longitudinal relaxation time; SE-EPI, spin echo-echo planar imaging; bFFE, balanced fast field echo; T_2_^*^, transverse relaxation time; ADC, Apparent Diffusion Coefficient; D, pure Diffusion coefficient; D^*^, pseudodiffusion coefficient; f_p_, perfusion fraction.

Table 2 shows the field strength dependence of longitudinal (T_1_) and transverse (T_2_^*^) relaxation times. As expected, T_1_-values are longer at 3 T compared with 1.5 T for both the SE-EPI and bFFE readout schemes. It should be noted that the “apparent” T_1_ measured using a bFFE readout scheme is shorter than the “true” T_1_ measured using a SE-EPI readout. The corticomedullary differentiation of T_1_ can be seen to be greater at 3 T compared with 1.5 T. Conversely, the transverse relaxation time (T_2_^*^) is longer at 1.5 T.

**Table 2:** Field strength variability in T_1_ and T_2_*/R_2_* in renal cortex and renal medulla, and corticomedullary differentiation (medulla-cortex) for healthy participants.

**Parameter**	**Field strength (T)**	**Renal cortex**		**Renal medulla**		**Corticomedullary differentiation (medulla-cortex)**	
		**Mean ± std.dev**.	**Number of subjects**	**Mean ± std.dev**.	**Number of subjects**	**Mean ± std.dev**.	**Number of subjects**
T_1_ SE-EPI	1.5	1,024 ± 71 ms	8	1,272 ± 140 ms	8	248 ± 68 ms	8
	3	1,367 ± 79 ms	21	1,655 ± 76 ms	20	286 ± 58 ms	20
T_1_ bFFE	1.5	1,053 ± 72 ms	58	1,318 ± 98 ms	38	265 ± 38 ms	38
	3	1,124 ± 114 ms	26	1,388 ± 126 ms	25	268 ± 80 ms	25
T_2_^*^	1.5	70.7 ± 2.4 ms	8	40.7 ± 2.8 ms	8	−30.0 ± 5.2 ms	8
	3	49.6 ± 6.6 ms	18	29.7 ± 5.4 ms	18	−19.9 ± 5.1 ms	18
R_2_^*^	1.5	14.2 ± 0.5 s^−1^	8	24.6 ± 1.7 s^−1^	2	10.5 ± 2.2 s^−1^	8
	3	20.6 ± 3.3 s^−1^	18	34.9 ± 6.9 s^−1^	18	14.3 ± 5.0 s^−1^	18

T_1_, longitudinal relaxation time; SE-EPI, spin echo-echo planar imaging; bFFE, balanced fast field echo; T_2_^*^, transverse relaxation time; R_2_^*^, transverse relaxation rate.

Table 3 provides the CoV and ICCs for the repeatability study at 3 T. The CoV is low for T_1_ (< 2.9%), ADC (2.9%), T_2_^*^ (4.1%), and kidney volume (4.2%). The ICCs were high for cortex perfusion (0.801), cortex and medulla T_1_ (0.848 and 0.997 using SE-EPI), renal artery flow (0.844) and total kidney volume (0.985).

**Table 3:** Intra subject repeatability for the multiparametric MRI measures in healthy participants.

**Parameter**		**Repeatability measures**			
		**CoV (%)**	**ICC**	**Number of subjects**	**Number of visits**
Single renal artery flow		14.4 ± 4.3	0.844	11	3
Single renal vein flow		18.8 ± 10.3	0.649	11	3
Total perfusion to single kidney		14.9 ± 3.8	0.611	10	3
Cortex perfusion		9.3 ± 4.4	0.801	11	3
Cortex T_1_(at 3 T)	SE-EPI	2.0 ± 1.5	0.848	9	2
	bFFE	2.3 ± 1.3	0.616	11	3
Medulla T_1_(at 3 T)	SE-EPI	1.8 ± 1.5	0.997	9	2
	bFFE	2.9 ± 2.4	0.239	11	3
Cortex T_2_^*^ (at 3 T)		4.1 ± 3.0	0.718	4	2
Cortex ADC		2.9 ± 2.0	0.745	10	3
Cortex D		9.5 ± 4.8	0.307	10	3
Cortex D^*^		38.8 ± 19.6	0.210	10	3
Cortex *f_*p*_*		21.5 ± 10.6	0.102	10	3
Total kidney volume		4.2 ± 2.6	0.985	11	3

CoV, coefficient of variation; ICC, intra class correlation; T_1_, longitudinal relaxation time; SE-EPI, spin echo-echo planar imaging; bFFE, balanced fast field echo; T_2_^*^, transverse relaxation time; ADC, apparent diffusion coefficient; D, pure diffusion coefficient; D^*^, pseudodiffusion coefficient; f_p_, perfusion fraction.

### Physiological modulation in healthy participants

**Figure 5:**
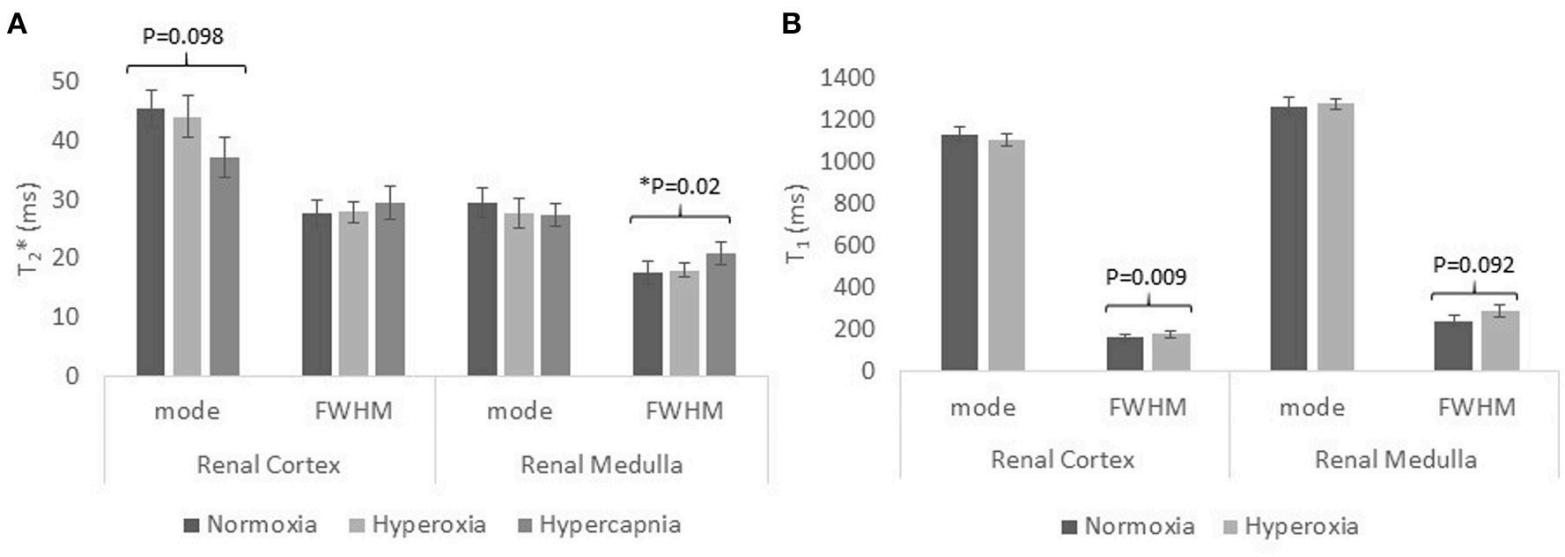
The mode and FWHM (full-width-at-half-maximum) in the renal cortex and medulla of healthy participants for **(A)** transverse relaxation time T_2_^*^ during normoxia, hyperoxia, and hypercapnia; **(B)** longitudinal relaxation time T_1_ during normoxia and hyperoxia.

Figure 5A shows the T_2_^*^ mode and FWHM in renal cortex and medulla at normoxia and during hypercapnia or during hyperoxia. During hypercapnia, there was a trend for a decrease in the T_2_^*^ mode in the renal cortex (*P* = 0.098, paired *t*-test), but the T_2_^*^ FWHM did not change. The T_2_^*^ mode in the renal medulla did not change, but the T_2_^*^ FWHM was found to increase (*P* = 0.02, paired *t*-test). During hyperoxia, there was no change in T_2_^*^ mode or FWHM in either the renal cortex or renal medulla.

Figure 5B shows the “apparent” T_1_ mode and FWHM for renal cortex and medulla at normoxia and during hyperoxia. During hyperoxia, there was no significant change in the “apparent” T_1_ mode of renal cortex or medulla, but the FWHM increased in the renal cortex (*P* = 0.009, paired *t*-test) and medulla (*P* = 0.092, paired *t*-test).

### Application in chronic kidney disease (CKD)

All 11 CKD patients had glomerular kidney disease, Table 4 provides demographic details of the patients and divides the healthy participants into young (<40 years) and older (>40 years) age groups for comparison. Table 5 provides the MRI results for each of these groups.

**Table 4:** Characteristics of healthy participants, split according to age, <40 years and >40 years, and the chronic kidney disease patient cohort.

	**Healthy participants <40 years**	**Healthy participants >40 years**	**CKD**
Male/Female	53/16	34/24	8/3
Age (years)	25 ± 4	60 ± 9	52 ± 14
Height (m)	1.76 ± 0.09	1.71 ± 0.09	1.74 ± 0.06
Weight (kg)	72.4 ± 10.0	76.0 ± 11.7	89.7 ± 10.5
BMI (kg/m^2^)	23 ± 2	26 ± 3	30 ± 4
eGFR (ml/min/1.73 m^2^)	–	–	51 ± 15
Systolic BP (mmHg)	–	–	132 ± 8
Diastolic BP (mmHg)	–	–	82 ± 8
Hypertension Medication yes/no	–	–	8/3

*CKD, Chronic Kidney Disease; BMI, body mass index; eGFR, estimated glomerular filtration rate; BP, blood pressure*.

**Table 5:** Multiparametric MRI measures in healthy participants split according to age and Chronic Kidney Disease patients.

**Parameter**		**Healthy participants<40 years**		**Healthy participants>40 years**		**CKD mean ± SD (*N* = 11)**	***P*****-value**		
		**Mean ± SD**	**N**	**Mean ± SD**	**N**		**ANOVA between groups**	**<40 years vs. >40 years**	**>40 years vs. CKD**
Renal artery flow (ml/min)		427 ± 117	33	329 ± 69	40	314 ± 148	0.0001	<0.0001	ns
Renal vein flow (ml/min)		437 ± 142	21	334 ± 76	7	212 ± 90	0.0002	0.0773	0.0134
Cortex perfusion (ml/100 g/min)		279 ± 75	42	232 ± 57	43	83 ± 68	<0.0001	0.0019	<0.0001
SE-EPI T_1_ (ms) (at 3 T)	Cortex	*1,347*±65	13	*1,399*±93	8	*1,530*±99	<0.0001	ns	0.0099
	Medulla	*1,635*±66	12	*1,685*±84	8	*1,726*±78	0.0254	ns	ns
	ΔT_1_	286 ± 28	12	286 ± 89	8	196 ± 45	0.0006	ns	0.0095
T_2_^*^ (ms) (at 3 T)	Cortex	48.9 ± 7.4	10	50.4 ± 5.8	8	54.6 ± 7.7	0.0860	ns	ns
	Medulla	29.8 ± 5.4	10	29.5 ± 5.7	8	33.0 ± 9.0	ns	ns	ns
Cortex ADC (×10^−3^ mm^2^/s)		2.3 ± 0.4	23	2.4 ± 0.2	16	2.1 ± 0.3	ns	ns	ns
Cortex D (×10^−3^ mm^2^/s)		1.7 ± 0.2	23	1.6 ± 0.4	15	1.8 ± 0.4	ns	ns	ns
Total kidney volume (ml)		361 ± 62	15	382 ± 51	7	409 ± 153	ns	ns	ns
Kidney volume, BSA corrected (ml/m^2^)		184 ± 29	15	190 ± 25	7	202 ± 86	ns	ns	ns

CKD, Chronic Kidney Disease; T_1_, longitudinal relaxation time; SE-EPI, spin echo-echo planar imaging; T_2_^*^, transverse relaxation time; ADC, apparent diffusion coefficient; D pure diffusion coefficient; BSA body surface area; ns, not significant.

Renal artery blood flow was significantly reduced in the older healthy participants compared to the young healthy participants, though no difference is seen between the older participants and CKD patients. In CKD, renal cortex perfusion and renal vein blood flow were lower than in older healthy participants. T_1_ SE-EPI in the renal cortex was significantly higher in CKD patients compared to older healthy participants, and corticomedullary T_1_ differentiation was reduced in CKD patients compared to older healthy participants. T_2_^*^ measured in the renal cortex and medulla was not significantly different in CKD patients compared with healthy participants. In this patient cohort, renal cortex ADC, D and total kidney volume in CKD patients were also not significantly different to healthy participants.

## Discussion

This article has demonstrated acquisition and analysis methods to perform multiparametric assessment of the kidneys in healthy participants and CKD patients.

### Variability, repeatability, and field strength dependence in healthy participants

We provide a comprehensive summary of MRI parameter values for healthy participants, results are in agreement with values reported across separate studies in the literature (Table 1). When comparing T_1_ measures for the renal cortex and medulla to literature values, it is important to consider the MR field strength and readout scheme used for the image acquisition. Here, we show the expected T_1_ increase with field strength (Table 2). Further, the computed T_1_ value is dependent on the image readout scheme, with a shorter “apparent” T_1_ measured for a bFFE readout compared to a SE-EPI readout, due to the influence of transverse relaxation on the bFFE readout. The T_1_ of the medulla was higher than that of the cortex, resulting in clearly visualized corticomedullary differentiation.

The CoV of T_1_ measures is very low, <3% for cortex and medulla (Table 3). Cutajar et al. reported CoVs of between 0.3 and 11.5% for repeatability of renal cortex T_1_ measures on the same day (Cutajar et al., 2012). Gillis et al. showed no significant difference between visits for repeated measures of renal cortex T_1_ using a MOLLI method (Gillis et al., 2014). However, MOLLI has some compromises, it is a cardiac gated scheme which provides poor sampling of the inversion recovery curve, requires a breath hold per slice, and since it uses a bFFE readout, its “apparent” T_1_ value is also affected by tissue fat content at 3 T (Mozes et al., 2016).

PC-MRI measures of renal artery and vein blood flow have a reasonably high CoV, as previously described (Bax et al., 2005; Khatir et al., 2014). This is likely a result of placement of the imaging slice. In contrast, ASL renal cortex perfusion is a voxel-wise measure and this is shown to have a low CoV and high ICC. Cutajar et al. reported CoVs of between 1.8 and 12.1% for repeatability of renal cortex perfusion measures on the same day (Cutajar et al., 2012) and Chowdhury et al. reported a within session CoV of 3.3% (Chowdhury et al., 2012), but to our knowledge there have been no CoVs reported for measures collected between visits. Gillis et al. showed no significant differences between visits for repeated measures of renal cortex perfusion (Gillis et al., 2014).

Thoeny et al. showed that the measured value of ADC is affected by the choice of b-values (Thoeny et al., 2005). Using only low b-values (0–100 s/mm^2^) will result in a high calculated ADC, whilst high b-values (500–1,000 s/mm^2^) will result in a low calculated ADC. Using a wide range of b-values provides the least variation in ADC between healthy participants. Here, we use b-values of between 0 and 500 s/mm^2^ and show comparable results to Thoeny et al. (2005). Cutajar et al. found no significant difference in ADC between sessions, their ADC values were higher than we report, likely due to their acquisition using only two b-values (Cutajar et al., 2011). The value of both ADC and D had a low CoV, whilst D^*^ and *f*_*p*_ had poor repeatability.

A wide range of renal cortex and medulla T_2_^*^ (R_2_^*^) values are reported in the literature. T_2_^*^ decreases with increasing field strength and is longer in the renal cortex compared to the renal medulla, indicating the hypoxic state of the medulla. The T_2_^*^/R_2_^*^ values we present are in agreement with several studies (Li et al., 2004a; Ding et al., 2013; Khatir et al., 2014; Piskunowicz et al., 2015; van der Bel et al., 2016), whilst others give lower (Simon-Zoula et al., 2006; Park et al., 2012) or higher (Li et al., 2004b) R_2_^*^-values. Khatir et al. (2014) measured similar between session CoVs to those we present here.

### Physiological modulations in healthy participants

Here, we assess the change in T_2_^*^ on hypercapnia and on hyperoxia, and the change in T_1_ in response to hyperoxia. T_2_^*^ and T_1_ relaxation times of tissues have been suggested to be a potential biomarker for renal tissue oxygenation (Jones et al., 2002; O'Connor et al., 2009; Winter et al., 2011; Donati et al., 2012; Khatir et al., 2014; Ganesh et al., 2016). Changes in T_2_^*^ arise from local field inhomogeneities created by deoxyhemoglobin (Hb) molecules. Increasing the inspired oxygen increases the ratio of diamagnetic oxyhemoglobin to paramagnetic deoxyhemoglobin (HbO_2_/Hb) leading to longer T_2_^*^. Increasing inspired carbon dioxide reduces the oxygen affinity of hemoglobin, thus leading to increases in the levels of deoxygenated Hb in venous blood and a reduction in T_2_^*^ (Milman et al., 2013). Changes in T_1_ arise from changes in levels of dissolved O_2_ in plasma and tissue, since oxygen is weakly paramagnetic (Young et al., 1981), thus increasing levels of oxygen acts to shorten T_1_.

There is discrepancy in the literature of the effect of breathing 100% oxygen on T_2_^*^. Some studies have shown no change in T_2_^*^ in the renal cortex (Jones et al., 2002; O'Connor et al., 2009; Khatir et al., 2014; Niendorf et al., 2015) or medulla (Jones et al., 2002), whilst a small number of studies show a small increase in T_2_^*^ in the renal cortex (Winter et al., 2011; Ganesh et al., 2016) and medulla (Donati et al., 2012; Khatir et al., 2014). At normal levels of inspired oxygen, the body maintains hemoglobin levels in arterial blood near to saturation level. During hyperoxia, a higher fraction of HbO_2_/Hb and a reduction in blood volume could both be expected to contribute to a small increase in T_2_^*^. As an alternative, hypercapnic-hyperoxia has been shown to cause a marked 50% increase in renal T_2_^*^-weighted signal intensity, suggesting this method provides enhanced sensitivity (Milman et al., 2013).

For T_1_, previous studies have shown a decrease in the renal cortex on breathing 100% oxygen, which is equivalent to ~600 mmHg (Jones et al., 2002; O'Connor et al., 2007, 2009; Ganesh et al., 2016). Here, we used our modified respiratory triggered scheme to measure T_1_ and independently controlled end-tidal concentrations of oxygen and carbon dioxide (constant to ~0.1 mmHg). No significant difference in the mode of T_1_ was found between hypercapnia and normoxia. The controlled gas delivery was equivalent to breathing ~80% oxygen, and this may explain the smaller T_1_ change seen in our data. It should be noted that breathing 100% oxygen can lead to hypocapnia (Becker et al., 1996) resulting in a reduction in flow.

To our knowledge, no studies of human kidneys have used hypercapnia. Winter et al. showed no change in T_2_^*^ at 1.5 T in the rabbit renal cortex when inspiring 10% carbon dioxide (balance air; Winter et al., 2011), whilst Ganesh et al. show a decrease in T_2_^*^ at 3 T when inspiring 10–30% carbon dioxide (21% oxygen, balance nitrogen; Ganesh et al., 2016). Milman et al. showed that hypercapnia induced by 5% CO_2_ inhalation caused a marked decline in hemodynamic response imaging maps, based on changes in the signal intensity of a T_2_^*^-weighted image, resembling results of studies in the liver (Milman et al., 2013). The level of inspired carbon dioxide in this work is significantly lower than 10%, this may explain why our T_2_^*^ decrease did not reach significance.

Alternative mechanisms of physiological modulation to assess renal oxygenation and microcirculation reactivity and functionality include water loading, sodium loading, or drug administration (e.g., angiotensin, furosemide, saline). Studies have shown that water loading results in an increase in BOLD T_2_^*^ in the medulla (Prasad et al., 1996; Prasad and Epstein, 1999; Tumkur et al., 2006a; Vivier et al., 2013; Ding et al., 2015), this is thought to be due to the production of endogenous prostaglandin PGE2 in the medulla which decreases deoxyhemoglobin (Hb) levels, but it is not possible to distinguish between changes in oxygen supply and oxygen consumption. Similar more pronounced results have been shown following administration of furosemide (a sodium pump inhibitor; Prasad et al., 1996; Li et al., 2004a; Tumkur et al., 2006b; Vivier et al., 2013), coupled with a larger increase in urinary output (Vivier et al., 2013). Interestingly, T_2_^*^ is not altered in older subjects after water loading (Prasad and Epstein, 1999) or furosemide administration (Epstein and Prasad, 2000).

### Chronic kidney disease

The standard clinical assessment of renal function is the estimated GFR (eGFR) calculated from serum creatinine concentration. However, this is a late marker of renal dysfunction, is often discordant with tissue damage, is subject to hemodynamic fluctuation, and cannot be used to assess individual kidney function. Kidney biopsy has sampling error associated with the small specimen size, and comes with associated risks of an invasive procedure. This pilot study has assessed the use of multiparametric MRI in CKD patients, potentially providing a number of techniques by which to assess kidney structure and function. Renal blood flow and renal cortex perfusion was lower in CKD patients compared with healthy participants. T_1_ values were increased in both renal cortex and medulla compared to healthy participants, though primarily in cortex, resulting in a loss of corticomedullary differentiation.

There have been a number of previous studies assessing changes in individual MRI parameters related to hemodynamics and structure in CKD patients (Inoue et al., 2011; Michaely et al., 2012; Xin-Long et al., 2012; Khatir et al., 2014, 2015; Milani et al., 2016). Studies have compared perfusion in CKD patients with healthy participants and found perfusion to be lower in CKD patients (Rossi et al., 2012; Tan et al., 2014). Gillis et al. showed that the T_1_ relaxation time was longer in CKD patients compared to healthy participants (Gillis et al., 2016). Further, ADC values have been shown to be reduced in CKD compared to healthy participants (Goyal et al., 2012). A recent study using DWI and T_1_ mapping has demonstrated changes in both kidney ADC and T_1_ in animal models and humans with CKD (Friedli et al., 2016). Prior studies have shown conflicting changes in measures of oxygenation in CKD, with some groups reporting a reduction in oxygenation in CKD, whilst others report no differences in cortical or medullary R_2_^*^ (Pruijm et al., 2014). Khatir et al. showed similar cortical and medulla R_2_^*^ values at baseline between patients and controls. But on inspiring 100% oxygen, R_2_^*^ significantly decreased in the renal cortex of CKD patients with no change in R_2_^*^ was observed in healthy participants. Medullary R_2_^*^ increased in both patients and controls on inspiring 100% oxygen (Khatir et al., 2015). Pruijm et al. (2014) assessed patients with CKD and arterial hypertension (Pruijm et al., 2014), no difference in R_2_^*^ was seen between the patient group and healthy participants at baseline. However, following administration of furosemide, a blunted R_2_^*^ decrease was seen in patients compared with healthy participants. Xin-Long et al. (2012) measured the corticomedullary differentiation in R_2_^*^ in healthy participants and CKD patients and found an increased differentiation in CKD patients compared to healthy participants (Xin-Long et al., 2012).

## Limitations

It is important to consider the different factors which can impact on reported MR measures. Inconsistent BOLD results have been widely documented between published studies, whilst Michaely et al. showed that in a study of 280 subjects, R_2_^*^ correlated poorly with eGFR (Michaely et al., 2012). This is likely since R_2_^*^ is only an estimator of oxygenation, and is confounded by many other factors, with it being suggested that changes in the blood volume fraction considerably influences renal T_2_^*^ (Niendorf et al., 2015). Estimates of total renal blood flow need to consider kidney volume to also compute total perfusion, and in CKD patients the shrinkage of the kidney should be considered, which can mean that blood flow per kidney is preserved. However, this correction does not take into account that the cortex and medulla may not lose volume at the same rate.

ICC's are high for some MRI parameters presented—T_1_, perfusion, renal artery flow and ADC—but other values are relatively low, presently hampering the introduction of these methods in clinical practice. Currently, MRI is expensive and multiple breath hold methods cannot be used in older, frail patients. Here, our multiparametric protocol includes a limited number of breath holds, with ASL, T_1_, and DWI data collected using respiratory triggered acquisitions. In this study, inter-observer variability was not assessed since the post-processing is automated, including ROI placement. Further automation of this pipeline could be included and with the introduction of greater processing power, maps could be computed online at the scanner. In future, functional sodium technology to provide information on renal concentrating capacity will provide a further additional measure for multiparametric protocols (Maril et al., 2006). At this point, studies showing that MRI parameters can predict hard outcomes, such as, end stage renal disease, death or rapid decline of kidney function are necessary. For ultimate use in the clinic, MRI protocols need to be time efficient, and so it will be important to define key MRI parameters of high ICC which can be used for clinical assessment.

## Conclusions

This paper has outlined a multiparametric MRI acquisition and analysis protocol for assessment of renal structure, hemodynamics and oxygenation. No other modality can combine non-invasive techniques to provide such a comprehensive evaluation of renal function as MRI. Studies showing that MRI has added value to simply monitoring serum creatinine and proteinuria in kidney disease, and that MRI can provide similar information as a kidney biopsy are now eagerly awaited. The ability of early identification of patients at risk of progressing to end-stage kidney disease and protocols to assess the efficacy of treatments would improve clinical outcome, be of cost benefit for society and improve life quality for the patients.

## Author contributions

EC: study design, acquisition, analysis and interpretation of data, statistical analysis, drafting of manuscript, final approval of manuscript, accountable for all aspects of the work; CEB: study design, acquisition, analysis and interpretation of data, critical revision of manuscript, final approval of manuscript, accountable for all aspects of the work; CRB: acquisition, analysis and interpretation of data, critical revision of manuscript, final approval of manuscript, accountable for all aspects of the work; BP: acquisition, analysis and interpretation of data, final approval of manuscript, accountable for all aspects of the work; HM: study design, interpretation of data, final approval of manuscript, accountable for all aspects of the work; MT: study design, interpretation of data, final approval of manuscript, accountable for all aspects of the work; NS: study design, interpretation of data, critical revision of manuscript, final approval of manuscript, accountable for all aspects of the work; SF: study design, acquisition, analysis and interpretation of data, drafting of manuscript, final approval of manuscript, accountable for all aspects of the work.

### Conflict of interest statement

The authors declare that the research was conducted in the absence of any commercial or financial relationships that could be construed as a potential conflict of interest.

## References

[B1] Adler S. Huang H. Wolin M. S. Kaminski P. M. (2004). Oxidant stress leads to impaired regulation of renal cortical oxygen consumption by nitric oxide in the aging kidney. J. Am. Soc. Nephrol. 15, 52–60. 10.1097/01.ASN.0000101032.21097.C5 14694157

[B2] Artz N. S. Sadowski E. A. Wentland A. L. Djamali A. Grist T. M. Seo S.. (2011a). Reproducibility of renal perfusion MR imaging in native and transplanted kidneys using non-contrast arterial spin labeling. J. Magn. Reson. Imaging 33, 1414–1421. 10.1002/jmri.22552 21591011 PMC3098463

[B3] Artz N. S. Sadowski E. A. Wentland A. L. Grist T. M. Seo S. Djamali A.. (2011b). Arterial spin labeling MRI for assessment of perfusion in native and transplanted kidneys. Magn. Reson. Imaging 29, 74–82. 10.1016/j.mri.2010.07.018 20850241 PMC3005910

[B4] Bax L. Bakker C. J. Klein W. M. Blanken N. Beutler J. J. Mali W. P. (2005). Renal blood flow measurements with use of phase-contrast magnetic resonance imaging: normal values and reproducibility. J. Vasc. Intervent. Radiol. 16, 807–814. 10.1097/01.RVI.0000161144.98350.28 15947044

[B5] Becker H. F. Polo O. McNamara S. G. Berthon-Jones M. Sullivan C. E. (1996). Effect of different levels of hyperoxia on breathing in healthy subjects. J. Appl. Physiol. 81, 1683–1690. 8904587 10.1152/jappl.1996.81.4.1683

[B6] Blantz R. C. Deng A. Miracle C. M. Thomson S. C. (2007). Regulation of kidney function and metabolism: a question of supply and demand. Trans. Am. Clin. Climatol. Assoc. 118, 23–43. 18528487 PMC1863590

[B7] Boss A. Martirosian P. Graf H. Claussen C. D. Schlemmer H. P. Schick F. (2005). High resolution MR perfusion imaging of the kidneys at 3 Tesla without administration of contrast media. RoFo 177, 1625–1630. 10.1055/s-2005-858761 16333784

[B8] Buchanan C. E. Cox E. F. Francis S. T. (eds.). (2015). Evaluation of Readout Schemes for Arterial Spin Labelling in the Human Kidney. Toronto, ON: Int Soc Mag Reson.

[B9] Buxton R. B. Frank L. R. Wong E. C. Siewert B. Warach S. Edelman R. R. (1998). A general kinetic model for quantitative perfusion imaging with arterial spin labeling. Magn. Reson. Med. 40, 383–396. 10.1002/mrm.1910400308 9727941

[B10] Chowdhury A. H. Cox E. F. Francis S. T. Lobo D. N. (2012). A randomized, controlled, double-blind crossover study on the effects of 2-L infusions of 0.9% saline and plasma-lyte(R) 148 on renal blood flow velocity and renal cortical tissue perfusion in healthy volunteers. Ann. Surg. 256, 18–24. 10.1097/SLA.0b013e318256be72 22580944

[B11] Cohen E. I. Kelly S. A. Edye M. Mitty H. A. Bromberg J. S. (2009). MRI estimation of total renal volume demonstrates significant association with healthy donor weight. Eur. J. Radiol. 71, 283–287. 10.1016/j.ejrad.2008.03.006 18436402

[B12] Cutajar M. Clayden J. D. Clark C. A. Gordon I. (2011). Test-retest reliability and repeatability of renal diffusion tensor MRI in healthy subjects. Eur. J. Radiol. 80, e263–e268. 10.1016/j.ejrad.2010.12.018 21227619

[B13] Cutajar M. Thomas D. L. Banks T. Clark C. A. Golay X. Gordon I. (2012). Repeatability of renal arterial spin labelling MRI in healthy subjects. Magma 25, 145–153. 10.1007/s10334-011-0300-9 22246289

[B14] Cutajar M. Thomas D. L. Hales P. W. Banks T. Clark C. A. Gordon I. (2014). Comparison of ASL and DCE MRI for the non-invasive measurement of renal blood flow: quantification and reproducibility. Eur. Radiol. 24, 1300–1308. 10.1007/s00330-014-3130-0 24599625

[B15] Dambreville S. Chapman A. B. Torres V. E. King B. F. Wallin A. K. Frakes D. H.. (2010). Renal arterial blood flow measurement by breath-held MRI: accuracy in phantom scans and reproducibility in healthy subjects. Magn. Reson. Med. 63, 940–950. 10.1002/mrm.22278 20373395 PMC3760266

[B16] de Bazelaire C. M. Duhamel G. D. Rofsky N. M. Alsop D. C. (2004). MR imaging relaxation times of abdominal and pelvic tissues measured *in vivo* at 3.0 T: preliminary results. Radiology 230, 652–659. 10.1148/radiol.2303021331 14990831

[B17] Debatin J. F. Ting R. H. Wegmuller H. Sommer F. G. Fredrickson J. O. Brosnan T. J.. (1994). Renal artery blood flow: quantitation with phase-contrast MR imaging with and without breath holding. Radiology 190, 371–378. 10.1148/radiology.190.2.8284383 8284383

[B18] Ding J. Xing W. Wu D. Chen J. Pan L. Sun J. (2015). Evaluation of renal oxygenation level changes after water loading using susceptibility-weighted imaging and T2^*^ mapping. Korean J. Radiol. 16, 827–834. 10.3348/kjr.2015.16.4.827 26175582 PMC4499547

[B19] Ding Y. Mason R. P. McColl R. W. Yuan Q. Hallac R. R. Sims R. D.. (2013). Simultaneous measurement of tissue oxygen level-dependent (TOLD) and blood oxygenation level-dependent (BOLD) effects in abdominal tissue oxygenation level studies. J. Magn. Reson. Imaging 38, 1230–1236. 10.1002/jmri.24006 23749420

[B20] Dobre M. C. Ugurbil K. Marjanska M. (2007). Determination of blood longitudinal relaxation time (T1) at high magnetic field strengths. Magn. Reson. Imaging 25, 733–735. 10.1016/j.mri.2006.10.020 17540286

[B21] Donati O. F. Nanz D. Serra A. L. Boss A. (2012). Quantitative BOLD response of the renal medulla to hyperoxic challenge at 1.5 T and 3.0 T. NMR Biomed. 25, 1133–1138. 10.1002/nbm.2781 22290729

[B22] Dong J. Yang L. Su T. Yang X. Chen B. Zhang J.. (2013). Quantitative assessment of acute kidney injury by noninvasive arterial spin labeling perfusion MRI: a pilot study. Sci. China Life Sci. 56, 745–750. 10.1007/s11427-013-4503-3 23740361

[B23] Epstein F. H. Prasad P. (2000). Effects of furosemide on medullary oxygenation in younger and older subjects. Kidney Int. 57, 2080–2083. 10.1046/j.1523-1755.2000.00057.x 10792627

[B24] Evans R. G. Gardiner B. S. Smith D. W. O'Connor P. M. (2008). Intrarenal oxygenation: unique challenges and the biophysical basis of homeostasis. Am. J. Physiol. Renal Physiol. 295, F1259–F1270. 10.1152/ajprenal.90230.2008 18550645

[B25] Fenchel M. Martirosian P. Langanke J. Giersch J. Miller S. Stauder N. I.. (2006). Perfusion MR imaging with FAIR true FISP spin labeling in patients with and without renal artery stenosis: initial experience. Radiology 238, 1013–1021. 10.1148/radiol.2382041623 16439565

[B26] Friedli I. Crowe L. A. Berchtold L. Moll S. Hadaya K. de Perrot T.. (2016). New Magnetic resonance imaging index for renal fibrosis assessment: a comparison between diffusion-weighted imaging and T1 mapping with histological validation. Sci. Rep. 6:30088. 10.1038/srep30088 27439482 PMC4954968

[B27] Ganesh T. Estrada M. Duffin J. Cheng H. L. (2016). T2^*^ and T1 assessment of abdominal tissue response to graded hypoxia and hypercapnia using a controlled gas mixing circuit for small animals. J. Magn. Reson. Imaging 44, 305–316. 10.1002/jmri.25169 26872559

[B28] Gardener A. G. Francis S. T. (2010). Multislice perfusion of the kidneys using parallel imaging: image acquisition and analysis strategies. Magn. Reson. Med. 63, 1627–1636. 10.1002/mrm.22387 20512866

[B29] Gillis K. A. McComb C. Foster J. E. Taylor A. H. Patel R. K. Morris S. T.. (2014). Inter-study reproducibility of arterial spin labelling magnetic resonance imaging for measurement of renal perfusion in healthy volunteers at 3 Tesla. BMC Nephrol. 15:23. 10.1186/1471-2369-15-23 24484613 PMC3909760

[B30] Gillis K. A. McComb C. Patel R. K. Stevens K. K. Schneider M. P. Radjenovic A.. (2016). Non-contrast renal magnetic resonance imaging to assess perfusion and corticomedullary differentiation in health and chronic kidney disease. Nephron 133, 183–192. 10.1159/000447601 27362585

[B31] Goyal A. Sharma R. Bhalla A. S. Gamanagatti S. Seth A. (2012). Diffusion-weighted MRI in assessment of renal dysfunction. Indian J. Radiol. Imaging 22, 155–159. 10.4103/0971-3026.107169 23599561 PMC3624736

[B32] Hammon M. Janka R. Siegl C. Seuss H. Grosso R. Martirosian P.. (2016). Reproducibility of kidney perfusion measurements with arterial spin labeling at 1.5 Tesla MRI combined with semiautomatic segmentation for differential cortical and medullary assessment. Medicine 95:e3083. 10.1097/MD.0000000000003083 26986143 PMC4839924

[B33] Heusch P. Wittsack H. J. Heusner T. Buchbender C. Quang M. N. Martirosian P.. (2013). Correlation of biexponential diffusion parameters with arterial spin-labeling perfusion MRI: results in transplanted kidneys. Invest. Radiol. 48, 140–144. 10.1097/RLI.0b013e318277bfe3 23249648

[B34] Hoad C. L. Palaniyappan N. Kaye P. Chernova Y. James M. W. Costigan C. (2015). A study of T(1) relaxation time as a measure of liver fibrosis and the influence of confounding histological factors. NMR Biomed. 28, 706–714. 10.1002/nbm.3299 25908098

[B35] Iles L. Pfluger H. Phrommintikul A. Cherayath J. Aksit P. Gupta S. N.. (2008). Evaluation of diffuse myocardial fibrosis in heart failure with cardiac magnetic resonance contrast-enhanced T1 mapping. J. Am. Coll. Cardiol. 52, 1574–1580. 10.1016/j.jacc.2008.06.049 19007595

[B36] Inoue T. Kozawa E. Okada H. Inukai K. Watanabe S. Kikuta T.. (2011). Noninvasive evaluation of kidney hypoxia and fibrosis using magnetic resonance imaging. J. Am. Soc. Nephrol. 22, 1429–1434. 10.1681/ASN.2010111143 21757771 PMC3148697

[B37] Jellis C. L. Kwon D. H. (2014). Myocardial T1 mapping: modalities and clinical applications. Cardiovasc. Diagn. Ther. 4, 126–137. 10.3978/j.issn.2223-3652.2013.09.03 24834410 PMC3996234

[B38] Jones R. A. Ries M. Moonen C. T. Grenier N. (2002). Imaging the changes in renal T1 induced by the inhalation of pure oxygen: a feasibility study. Magn. Reson. Med. 47, 728–735. 10.1002/mrm.10127 11948734

[B39] Karger N. Biederer J. Lusse S. Grimm J. Steffens J. Heller M.. (2000). Quantitation of renal perfusion using arterial spin labeling with FAIR-UFLARE. Magn. Reson. Imaging 18, 641–647. 10.1016/S0730-725X(00)00155-7 10930773

[B40] Khatir D. S. Pedersen M. Jespersen B. Buus N. H. (2014). Reproducibility of MRI renal artery blood flow and BOLD measurements in patients with chronic kidney disease and healthy controls. J. Magn. Reson. Imaging 40, 1091–1098. 10.1002/jmri.24446 24470349

[B41] Khatir D. S. Pedersen M. Jespersen B. Buus N. H. (2015). Evaluation of renal blood flow and oxygenation in CKD using magnetic resonance imaging. Am. J. Kidney Dis. 66, 402–411. 10.1053/j.ajkd.2014.11.022 25618188

[B42] Kiefer C. Schroth G. Gralla J. Diehm N. Baumgartner I. Husmann M. (2009). A feasibility study on model-based evaluation of kidney perfusion measured by means of FAIR prepared true-FISP arterial spin labeling (ASL) on a 3-T MR scanner. Acad. Radiol. 16, 79–87. 10.1016/j.acra.2008.04.024 19064215

[B43] Koh D. M. Collins D. J. Orton M. R. (2011). Intravoxel incoherent motion in body diffusion-weighted MRI: reality and challenges. Am. J. Roentgenol. 196, 1351–1361. 10.2214/AJR.10.5515 21606299

[B44] Le Bihan D. Breton E. Lallemand D. Aubin M. L. Vignaud J. Laval-Jeantet M. (1988). Separation of diffusion and perfusion in intravoxel incoherent motion MR imaging. Radiology 168, 497–505. 10.1148/radiology.168.2.3393671 3393671

[B45] Li L. P. Storey P. Pierchala L. Li W. Polzin J. Prasad P. (2004a). Evaluation of the reproducibility of intrarenal R2^*^ and DeltaR2^*^ measurements following administration of furosemide and during waterload. J. Magn. Reson. Imaging 19, 610–616. 10.1002/jmri.20043 15112311 PMC2915575

[B46] Li L. P. Vu A. T. Li B. S. Dunkle E. Prasad P. V. (2004b). Evaluation of intrarenal oxygenation by BOLD MRI at 3.0 T. J. Magn. Reson. Imaging 20, 901–904. 10.1002/jmri.20176 15503343 PMC2914562

[B47] Maril N. Margalit R. Rosen S. Heyman S. N. Degani H. (2006). Detection of evolving acute tubular necrosis with renal 23Na MRI: studies in rats. Kidney Int. 69, 765–768. 10.1038/sj.ki.5000152 16518333

[B48] Martirosian P. Klose U. Mader I. Schick F. (2004). FAIR true-FISP perfusion imaging of the kidneys. Magn. Reson. Med. 51, 353–361. 10.1002/mrm.10709 14755661

[B49] Michaely H. J. Metzger L. Haneder S. Hansmann J. Schoenberg S. O. Attenberger U. I. (2012). Renal BOLD-MRI does not reflect renal function in chronic kidney disease. Kidney Int. 81, 684–689. 10.1038/ki.2011.455 22237750

[B50] Michaely H. J. Schoenberg S. O. Ittrich C. Dikow R. Bock M. Guenther M. (2004). Renal disease: value of functional magnetic resonance imaging with flow and perfusion measurements. Invest. Radiol. 39, 698–705. 10.1097/00004424-200411000-00008 15486531

[B51] Milani B. Ansaloni A. Sousa-Guimaraes S. Vakilzadeh N. Piskunowicz M. Vogt B.. (2016). Reduction of cortical oxygenation in chronic kidney disease: evidence obtained with a new analysis method of blood oxygenation level-dependent magnetic resonance imaging. Nephrol. Dial. Transplant. [Epub ahead of print]. 10.1093/ndt/gfw362 27798200

[B52] Milman Z. Heyman S. N. Corchia N. Edrei Y. Axelrod J. H. Rosenberger C.. (2013). Hemodynamic response magnetic resonance imaging: application for renal hemodynamic characterization. Nephrol. Dial. Transplant. 28, 1150–1156. 10.1093/ndt/gfs541 23291364

[B53] Mozes F. E. Tunnicliffe E. M. Pavlides M. Robson M. D. (2016). Influence of fat on liver T1 measurements using modified Look-Locker inversion recovery (MOLLI) methods at 3T. J. Magn. Reson. Imaging 44, 105–111. 10.1002/jmri.25146 26762615 PMC4982078

[B54] Niendorf T. Pohlmann A. Arakelyan K. Flemming B. Cantow K. Hentschel J.. (2015). How bold is blood oxygenation level-dependent (BOLD) magnetic resonance imaging of the kidney? Opportunities, challenges and future directions. Acta Physiol. 213, 19–38. 10.1111/apha.12393 25204811

[B55] Niles D. J. Artz N. S. Djamali A. Sadowski E. A. Grist T. M. Fain S. B. (2016). Longitudinal assessment of renal perfusion and oxygenation in transplant donor-recipient pairs using arterial spin labeling and blood oxygen level-dependent magnetic resonance imaging. Invest. Radiol. 51, 113–120. 10.1097/RLI.0000000000000210 26561047 PMC4697870

[B56] Notohamiprodjo M. Chandarana H. Mikheev A. Rusinek H. Grinstead J. Feiweier T.. (2015). Combined intravoxel incoherent motion and diffusion tensor imaging of renal diffusion and flow anisotropy. Magn. Reson. Med. 73, 1526–1532. 10.1002/mrm.25245 24752998

[B57] O'Connor J. P. Jackson A. Buonaccorsi G. A. Buckley D. L. Roberts C. Watson Y.. (2007). Organ-specific effects of oxygen and carbogen gas inhalation on tissue longitudinal relaxation times. Magn. Reson. Med. 58, 490–496. 10.1002/mrm.21357 17763345

[B58] O'Connor J. P. Naish J. H. Jackson A. Waterton J. C. Watson Y. Cheung S. (2009). Comparison of normal tissue R1 and R2^*^ modulation by oxygen and carbogen. Magn. Reson. Med. 61, 75–83. 10.1002/mrm.21815 19097212

[B59] Park J. B. Santos J. M. Hargreaves B. A. Nayak K. S. Sommer G. Hu B. S.. (2005). Rapid measurement of renal artery blood flow with ungated spiral phase-contrast MRI. J. Magn. Reson. Imaging 21, 590–595. 10.1002/jmri.20325 15834919

[B60] Park S. H. Wang D. J. Duong T. Q. (2013). Balanced steady state free precession for arterial spin labeling MRI: initial experience for blood flow mapping in human brain, retina, and kidney. Magn. Reson. Imaging 31, 1044–1050. 10.1016/j.mri.2013.03.024 23664680 PMC3729883

[B61] Park S. Y. Kim C. K. Park B. K. Huh W. Kim S. J. Kim B. (2012). Evaluation of transplanted kidneys using blood oxygenation level-dependent MRI at 3 T: a preliminary study. Am. J. Roentgenol. 198, 1108–1114. 10.2214/AJR.11.7253 22528900

[B62] Piskunowicz M. Hofmann L. Zuercher E. Bassi I. Milani B. Stuber M.. (2015). A new technique with high reproducibility to estimate renal oxygenation using BOLD-MRI in chronic kidney disease. Magn. Reson. Imaging 33, 253–261. 10.1016/j.mri.2014.12.002 25523609

[B63] Prasad P. V. Epstein F. H. (1999). Changes in renal medullary pO2 during water diuresis as evaluated by blood oxygenation level-dependent magnetic resonance imaging: effects of aging and cyclooxygenase inhibition. Kidney Int. 55, 294–298. 10.1046/j.1523-1755.1999.00237.x 9893139 PMC2918873

[B64] Prasad P. V. Edelman R. R. Epstein F. H. (1996). Noninvasive evaluation of intrarenal oxygenation with BOLD MRI. Circulation 94, 3271–3275. 10.1161/01.CIR.94.12.3271 8989140

[B65] Pruijm M. Hofmann L. Piskunowicz M. Muller M. E. Zweiacker C. Bassi I.. (2014). Determinants of renal tissue oxygenation as measured with BOLD-MRI in chronic kidney disease and hypertension in humans. PLoS ONE 9:e95895. 10.1371/journal.pone.0095895 24760031 PMC3997480

[B66] Pruijm M. Milani B. Burnier M. (2017). Blood oxygenation level-dependent MRI to assess renal oxygenation in renal diseases: progresses and challenges. Front. Physiol. 7:667. 10.3389/fphys.2016.00667 28105019 PMC5214762

[B67] Ritt M. Janka R. Schneider M. P. Martirosian P. Hornegger J. Bautz W.. (2010). Measurement of kidney perfusion by magnetic resonance imaging: comparison of MRI with arterial spin labeling to para-aminohippuric acid plasma clearance in male subjects with metabolic syndrome. Nephrol. Dial. 25, 1126–1133. 10.1093/ndt/gfp639 19934080

[B68] Rossi C. Artunc F. Martirosian P. Schlemmer H. P. Schick F. Boss A. (2012). Histogram analysis of renal arterial spin labeling perfusion data reveals differences between volunteers and patients with mild chronic kidney disease. Invest. Radiol. 47, 490–496. 10.1097/RLI.0b013e318257063a 22766911

[B69] Schmitt P. Griswold M. A. Jakob P. M. Kotas M. Gulani V. Flentje M. (2004). Inversion recovery TrueFISP: quantification of T1, T2, and spin density. Magn. Reson. Med. 51, 661–667. 10.1002/mrm.20058 15065237

[B70] Schoenberg S. O. Just A. Bock M. Knopp M. V. Persson P. B. Kirchheim H. R. (1997). Noninvasive analysis of renal artery blood flow dynamics with MR cine phase-contrast flow measurements. Am. J. Physiol. 272, H2477–H2484. 9176319 10.1152/ajpheart.1997.272.5.H2477

[B71] Seuss H. Janka R. Prummer M. Cavallaro A. Hammon R. Theis R.. (2017). Development and evaluation of a semi-automated segmentation tool and a modified ellipsoid formula for volumetric analysis of the kidney in non-contrast T2-weighted MR images. J. Digit. Imaging 30, 244–254. 10.1007/s10278-016-9936-3 28025731 PMC5359215

[B72] Sigmund E. E. Vivier P. H. Sui D. Lamparello N. A. Tantillo K. Mikheev A.. (2012). Intravoxel incoherent motion and diffusion-tensor imaging in renal tissue under hydration and furosemide flow challenges. Radiology 263, 758–769. 10.1148/radiol.12111327 22523327

[B73] Simon-Zoula S. C. Hofmann L. Giger A. Vogt B. Vock P. Frey F. J.. (2006). Non-invasive monitoring of renal oxygenation using BOLD-MRI: a reproducibility study. NMR Biomed. 19, 84–89. 10.1002/nbm.1004 16411163

[B74] Skorecki K. Chertow G. M. Marsden P. A. Taal M. W. Yu A. S. L. (2016). Brenner and Rector's The Kidney, 10th Edn. Philadelphia, PA: Elsevier.

[B75] Steeden J. A. Muthurangu V. (2015). Investigating the limitations of single breath-hold renal artery blood flow measurements using spiral phase contrast MR with R-R interval averaging. J. Magn. Reson. Imaging 41, 1143–1149. 10.1002/jmri.24638 24723271

[B76] Suo S. Lin N. Wang H. Zhang L. Wang R. Zhang S.. (2015). Intravoxel incoherent motion diffusion-weighted MR imaging of breast cancer at 3.0 Tesla: comparison of different curve-fitting methods. J. Magn. Reson. Imaging 42, 362–370. 10.1002/jmri.24799 25407944

[B77] Tan H. Koktzoglou I. Prasad P. V. (2014). Renal perfusion imaging with two-dimensional navigator gated arterial spin labeling. Magn. Reson. Med. 71, 570–579. 10.1002/mrm.24692 23447145 PMC4429520

[B78] Thoeny H. C. De Keyzer F. Oyen R. H. Peeters R. R. (2005). Diffusion-weighted MR imaging of kidneys in healthy volunteers and patients with parenchymal diseases: initial experience. Radiology 235, 911–917. 10.1148/radiol.2353040554 15845792

[B79] Thomson S. C. Blantz R. C. (2008). Glomerulotubular balance, tubuloglomerular feedback, and salt homeostasis. J. Am. Soc. Nephrol. 19, 2272–2275. 10.1681/ASN.2007121326 18322161

[B80] Tumkur S. M. Vu A. T. Li L. P. Pierchala L. Prasad P. V. (2006a). Evaluation of intra-renal oxygenation during water diuresis: a time-resolved study using BOLD MRI. Kidney Int. 70, 139–143. 10.1038/sj.ki.5000347 16572109 PMC2919062

[B81] Tumkur S. Vu A. Li L. Prasad P. V. (2006b). Evaluation of intrarenal oxygenation at 3.0 T using 3-dimensional multiple gradient-recalled echo sequence. Invest. Radiol. 41, 181–184. 10.1097/01.rli.0000187166.43871.fb 16428990 PMC2915892

[B82] Tunnicliffe E. M. Banerjee R. Pavlides M. Neubauer S. Robson M. D. (2017). A model for hepatic fibrosis: the competing effects of cell loss and iron on shortened modified Look-Locker inversion recovery T1 (shMOLLI-T1) in the liver. J. Magn. Reson. Imaging 45, 450–462. 10.1002/jmri.25392 27448630

[B83] van den Dool S. W. Wasser M. N. de Fijter J. W. Hoekstra J. van der Geest R. J. (2005). Functional renal volume: quantitative analysis at gadolinium-enhanced MR angiography–feasibility study in healthy potential kidney donors. Radiology 236, 189–195. 10.1148/radiol.2361021463 15987973

[B84] van der Bel R. Coolen B. F. Nederveen A. J. Potters W. V. Verberne H. J. Vogt L.. (2016). Magnetic resonance imaging-derived renal oxygenation and perfusion during continuous, steady-state angiotensin-II infusion in healthy humans. J. Am. Heart Assoc. 5:e003185. 10.1161/JAHA.115.003185 27021686 PMC4943284

[B85] Venkatachalam M. A. Griffin K. A. Lan R. Geng H. Saikumar P. Bidani A. K. (2010). Acute kidney injury: a springboard for progression in chronic kidney disease. Am. J. Physiol. Renal Physiol. 298, F1078–F1094. 10.1152/ajprenal.00017.2010 20200097 PMC2867413

[B86] Vivier P. H. Storey P. Chandarana H. Yamamoto A. Tantillo K. Khan U. (2013). Renal blood oxygenation level-dependent imaging: contribution of R2 to R2^*^ values. Invest. Radiol. 48, 501–508. 10.1097/RLI.0b013e3182823591 23385400 PMC5053024

[B87] Wang J. Zhang Y. Yang X. Wang X. Zhang J. Fang J.. (2012). Hemodynamic effects of furosemide on renal perfusion as evaluated by ASL-MRI. Acad. Radiol. 19, 1194–1200. 10.1016/j.acra.2012.04.021 22958717

[B88] Winter J. D. Estrada M. Cheng H. L. (2011). Normal tissue quantitative T1 and T2^*^ MRI relaxation time responses to hypercapnic and hyperoxic gases. Acad. Radiol. 18, 1159–1167. 10.1016/j.acra.2011.04.016 21704536

[B89] Wittsack H. J. Lanzman R. S. Mathys C. Janssen H. Modder U. Blondin D. (2010). Statistical evaluation of diffusion-weighted imaging of the human kidney. Magn. Reson. Med. 64, 616–622. 10.1002/mrm.22436 20665805

[B90] Xin-Long P. Jing-Xia X. Jian-Yu L. Song W. Xin-Kui T. (2012). A preliminary study of blood-oxygen-level-dependent MRI in patients with chronic kidney disease. Magn. Reson. Imaging 30, 330–335. 10.1016/j.mri.2011.10.003 22244540

[B91] Young I. R. Clarke G. J. Bailes D. R. Pennock J. M. Doyle F. H. Bydder G. M. (1981). Enhancement of relaxation rate with paramagnetic contrast agents in NMR imaging. J. Comput. Tomogr. 5, 543–547. 10.1016/0149-936X(81)90089-8 7053127

[B92] Zhang J. L. Sigmund E. E. Chandarana H. Rusinek H. Chen Q. Vivier P. H.. (2010). Variability of renal apparent diffusion coefficients: limitations of the monoexponential model for diffusion quantification. Radiology 254, 783–792. 10.1148/radiol.09090891 20089719 PMC2851010

